# Afghanistan's Ethnic Groups Share a Y-Chromosomal Heritage Structured by Historical Events

**DOI:** 10.1371/journal.pone.0034288

**Published:** 2012-03-28

**Authors:** Marc Haber, Daniel E. Platt, Maziar Ashrafian Bonab, Sonia C. Youhanna, David F. Soria-Hernanz, Begoña Martínez-Cruz, Bouchra Douaihy, Michella Ghassibe-Sabbagh, Hoshang Rafatpanah, Mohsen Ghanbari, John Whale, Oleg Balanovsky, R. Spencer Wells, David Comas, Chris Tyler-Smith, Pierre A. Zalloua, The Genographic Consortium

**Affiliations:** 1 The Lebanese American University, Chouran, Beirut, Lebanon; 2 Evolutionary Biology Institute, Pompeu Fabra University, Barcelona, Spain; 3 Bioinformatics and Pattern Discovery, IBM T. J. Watson Research Centre, Yorktown Heights, New York, United States of America; 4 Biological Sciences, School of Biological Sciences, University of Portsmouth, Portsmouth, United Kingdom; 5 Mashhad University of Medical Sciences, Mashhad, Iran; 6 Research Centre for Medical Genetics, Russian Academy of Medical Sciences, Moscow, Russia; 7 The Genographic Project, National Geographic Society, Washington, D.C., United States of America; 8 Wellcome Trust Genome Campus, The Wellcome Trust Sanger Institute, Hinxton, Cambridgeshire, United Kingdom; 9 Harvard School of Public Health, Harvard University, Boston, Massachusetts, United States of America; Erasmus University Medical Center, The Netherlands

## Abstract

Afghanistan has held a strategic position throughout history. It has been inhabited since the Paleolithic and later became a crossroad for expanding civilizations and empires. Afghanistan's location, history, and diverse ethnic groups present a unique opportunity to explore how nations and ethnic groups emerged, and how major cultural evolutions and technological developments in human history have influenced modern population structures. In this study we have analyzed, for the first time, the four major ethnic groups in present-day Afghanistan: Hazara, Pashtun, Tajik, and Uzbek, using 52 binary markers and 19 short tandem repeats on the non-recombinant segment of the Y-chromosome. A total of 204 Afghan samples were investigated along with more than 8,500 samples from surrounding populations important to Afghanistan's history through migrations and conquests, including Iranians, Greeks, Indians, Middle Easterners, East Europeans, and East Asians. Our results suggest that all current Afghans largely share a heritage derived from a common unstructured ancestral population that could have emerged during the Neolithic revolution and the formation of the first farming communities. Our results also indicate that inter-Afghan differentiation started during the Bronze Age, probably driven by the formation of the first civilizations in the region. Later migrations and invasions into the region have been assimilated differentially among the ethnic groups, increasing inter-population genetic differences, and giving the Afghans a unique genetic diversity in Central Asia.

## Introduction

Afghanistan is a landlocked country at the intersection of Central Asia, South Asia, and the Middle East that has held a strategic position throughout history. It was a crossroad of ancient trade routes and human migrations. The main east-west trade routes passed through its northern and southern plains, and through its mountain passes before the ascendancy of waterborne trade between Europe and the Far East.

Paleolithic humans probably inhabited the caves of Afghanistan as long as 50,000 years ago (ya). In northern Afghanistan, flake tools found in Dara Dadil, Darra Chakhmakh, and elsewhere indicate the probable existence of Middle Paleolithic industries [1]. Northern Afghanistan also sits in a region of the development of the earliest agricultural communities, marked by domestication of the wheat/barley, sheep/goat/cattle complex leading to the Neolithic revolution (10,000–7,000 ya), later supporting the economy of early urban Bronze Age civilizations in Central Asia at the Bactria-Margiana Archaeological Complex (4300–3700 ya) and in India at the Indus Valley (5300–3800 ya) [2]. It has been proposed that the decline of these early civilizations was accompanied by, or was the result of, the expanding populations from the Eurasian steppe, reaching the Indian subcontinent in the late Harappan period [3].

The second and first millennia BCE were also marked by the influx of Iranian tribes, later ruling Afghanistan as part of the Achaemenid Empire established by Cyrus the Great (550 BCE) [4]. The military might of the Achaemenids was destroyed by Alexander the Great, bringing Hellenic language and culture to the region. During the next several centuries, control over Afghanistan was contested among the Seleucids, Bactrians, Parthians, and Indians of the Mauryan dynasty [5]. The first century CE, brought a new invasion of Iranian tribes under the leadership of the Kushan tribes, who adopted and spread Buddhism. After they have conquered most of Persia, Arabic armies invaded Afghanistan spreading Islam. Mongol and Turco-Mongol expansions brought turmoil to the region, marked by periods of instability to the Silk Road traffic [4], which was later reduced permanently with the establishment of European maritime trade systems.

The present population of Afghanistan contains many diverse elements, the result of large-scale migrations and conquests that influenced its culture and demography. Pashtuns are the largest ethnic group in Afghanistan, accounting for about 42 percent of the population, with Tajiks (27%), Hazaras (9%), Uzbeks (9%), Aimaqs (4%), Turkmen people (3%), Baluch (2%), and other groups (4%) making up the remainder [6]. In the present study, eight ethnic groups were examined, with a focus on the largest four groups: - The Pashtuns, traditionally lived a seminomadic lifestyle, they reside mainly in southern and eastern Afghanistan and in western Pakistan. They speak Pashto which is a member of the Eastern Iranian languages. - The Tajiks are a Persian-speaking ethnic group which are closely related to the Persians of Iran. In Afghanistan, they are the largest Tajik population outside their homeland to the north in Tajikistan. - The Hazara population speaks Persian with some Mongolian words. They believe they are descendants of Genghis Khan's army that invaded during the twelfth century. - The Uzbeks are a Turkic speaking group that have been living a sedentary farming lifestyle in Northern Afghanistan.

While previous theories about the origin of the Afghans are usually based on oral traditions or scanty historical information (Table S1), few studies have explored the genetic structure of the Afghan people, and those that did were limited to either listing of autosomal short tandem repeats (STRs) frequencies [7], [8] or Y-chromosome STR analysis in a single ethnic group [9]. In this study, we present an extensive analysis of the Y-chromosomal variation in the major ethnic groups of Afghanistan. We provide, for the first time, deep phylogenetic information on Afghan haplogroup memberships, and we also analyze 19 Y-chromosomal STRs allowing fine comparisons across and among populations. We use this information to explore whether the ethnic groups in Afghanistan reflect different social systems that arose in a common population or whether cultural differences are founded on already existing genetic differences. We also seek to understand the genetic composition of modern Afghans in the context of surrounding populations as well as other possible source populations, identifying traces of historical movements that influenced the different ethnic groups, and exploring how the establishment of the first civilizations in the region affected the present Afghan genetic diversity.

## Materials and Methods

### Ethics Statement

All participants recruited and genotyped in the present study had at least three generations of paternal ancestry in their country of birth, and provided details of their geographical origin and written consent for this study, which was approved by the IRB of the Lebanese American University.

### Subjects and Comparative Datasets

The modern populations selected for this study were those from regions with ancient historical importance to Afghanistan through conquest or migration, including Iranians, Greeks and Indians, in addition to populations with more recent impacts, such as the Arab expansion in the 7th century and the East Asian invasions in the 13^th^ and 14^th^ century. In addition, we have also included populations from the Pontic-Caspian steppe region, from West Russia and East Europe, which were possibly involved in the Indo-European migrations that reached the Iranian plateau and Northern India.

A total of 8,706 samples were used in the analyses including 204 newly genotyped samples from Afghanistan. The genotyping results and the subjects' paternal province and their city or village of origin when available are listed in Table S2. The dataset used include Middle Easterns (2,720 samples) [10], [11], [12], [13], [14], Central/South Asians (1,335 samples) [15], [16], [17], [18], East Asians (1,029 samples) [15], [19], Caucasians (1,525 samples) [20], West Russians (545 samples) [21], Europeans (1,123 samples) [21], [22], [23], [24], [25], and Africans (222 samples) [26], [27]. More details on the analyzed samples are listed in Table S3.

### Genotyping

DNA was extracted from blood or buccal swabs using a standard phenol-chloroform protocol. Samples were genotyped using the Applied Biosystems 7900HT Fast Real-Time PCR System with a set of 52, highly informative, custom Y-chromosomal binary marker assays (Applied Biosystems, Foster City, CA) from the non-recombining portion of the Y chromosome which define 32 different haplogroups. A total of 19 Y-chromosome STR loci were analyzed for each sample in two multiplexes on an Applied Biosystems 3130xl Genetic Analyzer. The first multiplex contained the standard 17 loci of the Y-filer™ PCR Amplification kit (Applied Biosystems, Foster City, CA). The remaining two loci, DYS388 and DYS426, were genotyped in a custom multiplex. STR alleles were named according to previous recommendations [28].

### Statistical Analyses

#### Haplogroup Frequencies and Principal Component Analysis

Fisher's exact tests were performed on haplogroups vs populations to identify which haplogroups were significantly over- or under- represented in Afghanistan's ethnic groups. A principal component analysis (PCA) [29], was performed on relative haplogroup frequencies normalized within populations, centered, and without variance normalization. Since haplogroup resolution was not uniform across studies, the haplogroups were reduced to the most informative derived markers shared across studies.

#### Genetic Distances, Multidimensional Scaling and Barrier Analysis

Non-metric multidimensional scaling (MDS) [30] was performed using Φ*_ST_* distances between populations computed by ARLEQUIN [31] on Y-STR loci DYS19, DYS389I, DYS389b, DYS390, DYS391, DYS392, DYS393, DYS437, DYS438, DYS439, DYS448, DYS456, DYS458, DYS635, GATA H4.

Monmonier's maximum difference algorithm [32] was implemented using Barrier [33]. The algorithm enables interpretation of microevolutionary processes in a geographic context, identifying genetic barriers that can be visualized on a map.

#### AMOVA

Significance of population structures created by Barrier was tested using AMOVA [34], implemented in ARLEQUIN [31]. We also tested whether geography or Barrier structures better explained the present distribution of diversity. AMOVA seeks to identify variance within populations due to drift by comparing variation among groups of similar populations via a nested analysis of variance. First, populations were grouped according to their geographic location as follows; 1- Afghanistan: Pashtun, Tajik, Uzbek, Hazara. 2- East Europe: Belarus, West Russia. 3- Caucasus: Avar, Darginian, Lezgi, Abkhazian, Circassian. 4- Middle East and Europe: Greece, Turkey, Lebanon, Syria. 5- Iran: East Azerbaijan, Markazi, Mazandaran, Qazvin, Sistan and Baluchistan. 6- India: North, West, South.

Second, populations were grouped according to the identified barriers; 1- Pashtun, Tajik, North India, West India. 2- Hazara, Uzbek 3- Caucasus: Avar, Darginian, Lezgi. 4- Caucasus: Circassian, Abkhazian, 5- Iran: East Azerbaijan, Markazi, Mazandaran, Qazvin, Sistan and Baluchistan. 6- Belarus, West Russia. 7- Middle East and Europe: Greece, Turkey, Lebanon, Syria.

#### Reduced Median Networks

Reduced Median (RM) Networks [35] of STR haplotypes within C-M130, R1a1a-M17, E1b1b1-M35, and B-M60 were calculated using a reduction threshold of 1, with no STR weighting.

#### BATWING

We applied BATWING [36] to compute candidate population splits in the modal tree among regional populations within and around Afghanistan in order to test whether BARRIER-identified population separations also showed older splits, exploring multiple combinations of populations. The Hastings-Metropolis algorithm will tend to select larger likelihoods for the leading genetic support assuming all the populations originally emerged from one population with no genetic flow subsequent to each splitting event. This provides a very specific view in determining genetic relationships among the populations which could be compared and contrasted with other methods, such as MDS or BARRIER [33]. STRs used were those described under the MDS section above.

The mutation rate priors applied to these calculations were those proposed in Xue et al. [19] based on Zhivotovsky et al.'s rate estimates [37]. There are differences between mutation rates that appear to accumulate over multiple generations (an “evolutionary rate”) versus those that accumulate from generation to generation (a “genealogical rate”) [38], which appears yet unresolved. Nevertheless, the topology of the population splits BATWING predicts, and the relative periods of isolation are proportionately unaffected. Therefore, the population split trees still serve for comparison with BARRIER and other methods regardless of the mutation rate. Effective population sizes tend to scale inversely with the rates, with a slight impact due to the effective population size prior. Use of the Zhivotovsky rates in prior publications allows for comparisons with other publications that applied the same rates.

The data were partitioned into multiple runs (Table S6). The independent computation of multiple trees with different subsets and groupings of populations should produce similar population splits and ages of population divisions among configurations. One caveat is that inclusion of other populations may provide more support to different candidate modal trees. Therefore, comparisons among multiple runs provide a consistency check for convergence and stability: each of the runs must correspond with the others at the points of their shared topologies. Given agreement between BATWING runs, a composite tree comprised of these multiple runs, and connected through shared branches, can be constructed.

The Indian populations structures resulted in slower equilibration than was seen among the other populations. After equilibration, the Indian populations showed older splits among them than is shown between India as a whole and the other populations when India is pooled. This older split may have resulted partly from differences in weights among candidate trees that the Metropolis-Hastings algorithm samples based on the likelihood ratios derived from the population configurations that will lead to different modal trees with different split times. Alternatively, the older split may have also resulted from violations of the assumption of isolation after population splitting. These complications led to the separate treatments of India BATWING runs from the western populations runs.

## Results

Genotyping revealed 32 halpogroups present in Afghanistan's ethnic groups among our samples. Haplogroups R1a1a-M17, C3-M217, J2-M172, and L-M20 were the most frequent when Afghan ethnic groups were pooled, together comprising >66% of the chromosomes. Absolute and relative haplogroup frequencies are tabulated in Table S4.

Haplogroup frequencies across the major ethnic groups revealed large differences. In particular, frequencies of haplogroup C3-M217, which is mainly found in East Asia, and haplogroup R1a1a-M17, which is found in Eurasia, varied substantially among the Afghan groups. C3-M217 was significantly more frequent (p = 4.55×10^−9^) in Uzbeks (41.18%) and Hazaras (33.33%) than it was in Tajiks (3.57%) and Pashtuns (2.04%). On the other hand, R1a1a-M17 was significantly more frequent (p = 3.00×10^−6^) in Pashtuns (51.02%) and Tajiks (30.36%) than in Uzbeks (17.65%) and Hazaras (6.67%). RM networks of C3-M217 (Figure S1A) and R1a1a-M17 (Figure S1B) show that when a haplogroup was infrequent in an ethnic group, its haplotypes existed on branches not shared with other Afghans, suggesting that the underrepresented haplogroups are not the result of a gene flow between the ethnic groups, but probably a direct assimilation from source populations.

Haplogroups autochthonous to India [15]; L-M20, H-M69, and R2a-M124 were found more (p = 0.004) in Pashtuns (20.41%) and Tajiks (19.64%) than in Uzbeks (5.88%) and Hazaras (5%). E1b1b1-M35 was found in Hazaras (5%) and Uzbeks (5.88%) but not in Pashtuns and Tajiks. RM network of E1b1b1-M35 (Figure S1C) shows that Afghanistan's lineages are correlated with Middle Easterners and Iranians. We also note the presence of the African B-M60 only in Hazara, with a relatively recent common founder ancestor from East Africa as shown in the RM network (Figure S1D).

**Figure 1:**
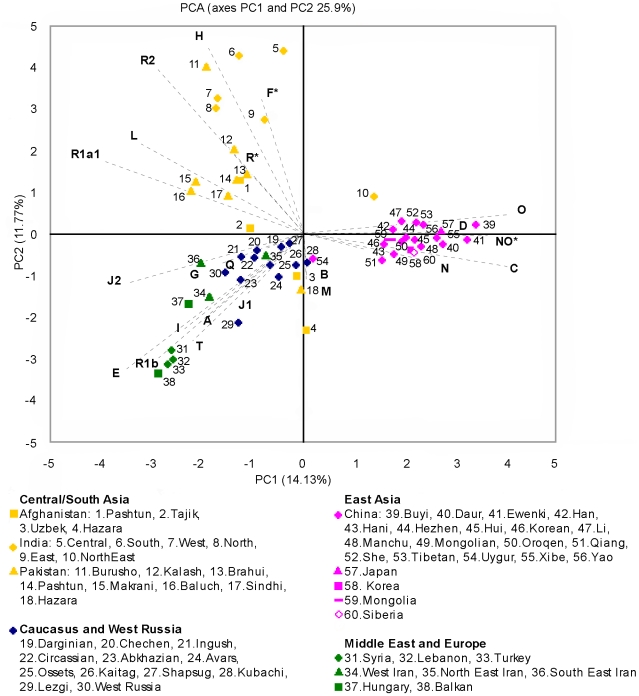
PCA derived from Y-chromosomal haplogroup frequencies. The two leading principal components display the variance. The superimposed biplot shows the contribution of each haplogroup as grey component loading vectors.

PCA of the haplogroups frequency (Figure 1) also shows differences among Afghans. Although the worldwide populations are mostly clustered according to geography, Afghan groups appear to show more affinity to non-Afghans than to each others. Pashtun and Hazara in Afghanistan and Pakistan show affinity to their ethnic groups across borders. The Afghan Tajiks show equal distance to Central Asia and to Iran/Caucasus/West Russia. The Afghan Hazara, Afghan Uzbek, and Pakistan Hazara sit between East Asia and the Middle East/Europe-Caucasus/West Russia cluster.

**Figure 2:**
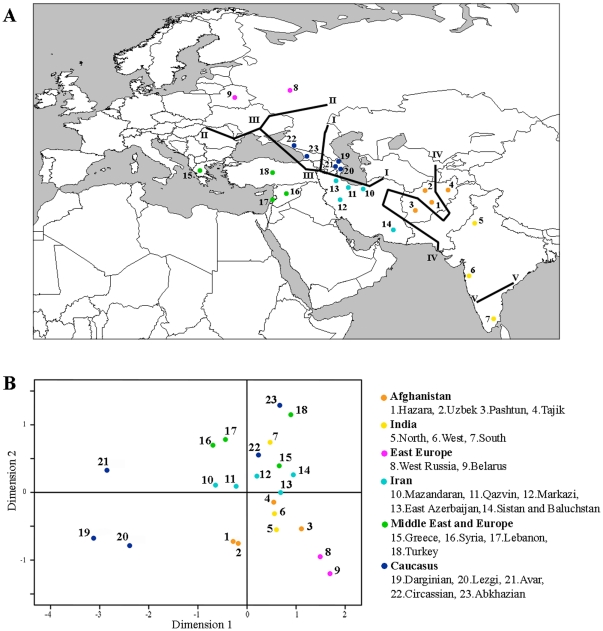
Population genetic structure vs geography. Genetic barriers (A) and MDS plot (B) based on the Φ*_ST_*'s distances between populations derived from Y-STR data.

More details about the structure of the Afghan population appear in the MDS of the 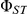
's (Figure 2B) which shows that the Afghan Pashtun and Tajik are closer to North and West Indians than to the other Afghans; Hazara and Uzbek. This cluster also sits between East Europeans and Iranians more close to the Iranians especially to East Azerbaijan. Furthermore, Barrier (Figure 2A) shows that Barrier IV splits the Afghan populations separating the Hazara and Uzbek from the Pashtun, Tajik and the Indian populations, creating groups of populations that have less variation within the groups (2.30%, p<0.001) and more variation among groups (10.48%, p<0.001) compared to populations grouped by region or country (within groups = 4.95%, p<0.001, among groups = 7.16%, p<0.001) (Table S5).

**Figure 3:**
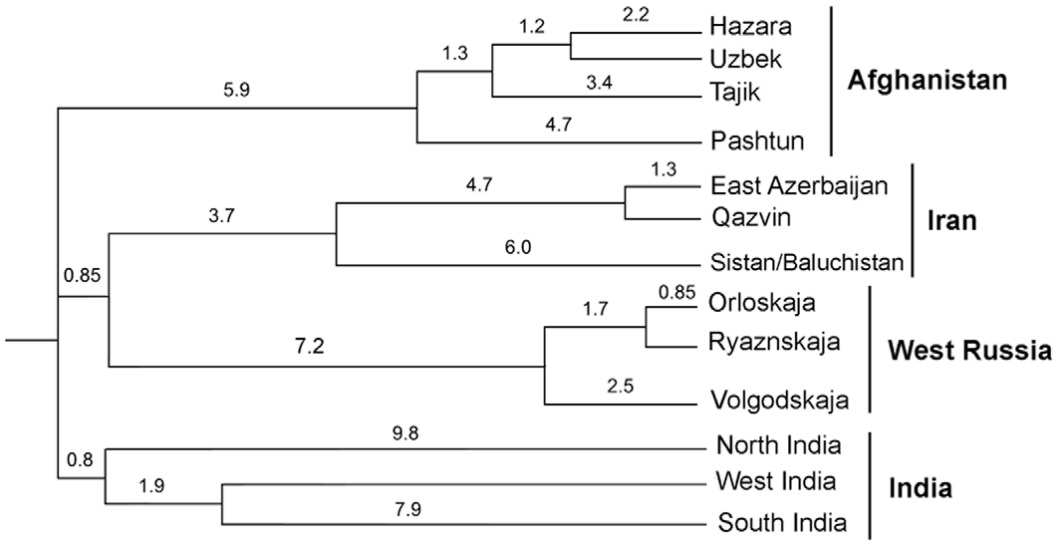
Composite BATWING population splitting. The composite tree is constructed from data sets described in the text, based on the results displayed in Table S6, with a pruned leading topology and averaged times. Numbers indicate branch lengths measured in thousand years.

To explore the time depth in which the above reported structures have emerged, we employed BATWING to create hypotheses on historical population splitting and coalescent events, reflecting dominating genetic ancestral structures identified in BATWING's modal trees from which the current populations have emerged (Table S6). The BATWING results showed that most of the regional splits occurred around 10 kya (95% CI 7,100–15,825) (Figure 3). These splits coincide with post LGM expansions that have led to the Neolithic agricultural revolution. During this period Afghans, Iranians, Indians and East Europeans most likely emerged as distinct unstructured populations. BATWING showed another wave of splits that started later and may have created the inter-population structures. This second wave of splits started in Afghans 4.7 kya (95% CI 2,775–7,725), marking the start of civilization building and displacements, and these splits appear to have continued to nearly modern times. BATWING results in general corroborated the geographical splits identified by BARRIER.

## Discussion

This study describes for the first time the Y-chromosome diversity of the main ethnic groups in Afghanistan. We have explored the genetic composition of modern Afghans and correlated their genetic diversity with well established historical events and movements of neighboring populations. The study data strongly shows that continuous migrations and movements through Central Asia since at least the Holocene, have created populations structures that today, are highly correlated with ethnicity in Afghanistan.

A previous study on Pakistan [39], that included ethnic groups also present in Afghanistan (Baluch, Hazara, Pashtun), showed that Y-chromosome variation was structured by geography and not by ethnic affiliation. With the exception of Hazara, all ethnic groups in Pakistan were shown to have similar Y-chromosome diversity, they clustered with South Asians, and they are close to Middle Eastern males. A Y-chromosome study [40] on populations from Turkmenistan, Uzbekistan, Kazakhstan, Kyrgyzstan, and Tajikstan, found that there is greater diversity among populations that share the same ethnic group than among the ethnic groups themselves. These observations support a common genetic ancestry hypothesis for these populations irrespective of ethnicity. We have also found substantial differences among the various groups of Afghanistan. The inter-ethnic comparisons however could not be tested in this study since information on tribe and clan affiliation was not available. The high genetic diversity observed among Afghanistan's groups has also been observed in other populations of Central Asia [41], [42], [43], [44], [45]. It is possibly due to the strategic location of this region and its unique harsh geography of mountains, deserts and steppes, which could have facilitated the establishment of social organizations within expanding populations, and helped maintaining genetic boundaries among groups that have developed over time into distinct ethnicities.

The RM networks of the major common haplogroups show that the flow of paternal lineages among the various ethnic groups is very limited, and it is consistent with high level of endogamy practiced by these groups. Similar Y-chromosome results have been previously reported among the Central Asian ethnic groups [40], but with less pronounced genetic differentiation in maternal lineages [40], most likely the results of endogamous practices that were tolerant to assimilation of foreign females.

The prevailing Y-chromosome lineage in Pashtun and Tajik (R1a1a-M17), has the highest observed diversity among populations of the Indus Valley [46]. R1a1a-M17 diversity declines toward the Pontic-Caspian steppe where the mid-Holocene R1a1a7-M458 sublineage is dominant [46]. R1a1a7-M458 was absent in Afghanistan, suggesting that R1a1a-M17 does not support, as previously thought [47], expansions from the Pontic Steppe [3], bringing the Indo-European languages to Central Asia and India.

MDS and Barrier analysis have identified a significant affinity between Pashtun, Tajik, North Indian, and West Indian populations, creating an Afghan-Indian population structure that excludes the Hazaras, Uzbeks, and the South Indian Dravidian speakers. In addition, gene flow to Afghanistan from India marked by Indian lineages, L-M20, H-M69, and R2a-M124, also seems to mostly involve Pashtuns and Tajiks. This genetic affinity and gene flow suggests interactions that could have existed since at least the establishment of the region's first civilizations at the Indus Valley and the Bactria-Margiana Archaeological Complex.

Furthermore, BATWING results indicate that the Afghan populations split from Iranians, Indians and East Europeans at about 10.6 kya (95% CI 7,100–15,825), which marks the start of the Neolithic revolution and the establishment of the farming communities. In addition, Pashtun split first from the rest of the Afghans around 4.7 kya (95% CI 2,775–7,725), which is a date marked by the rise of the Bronze Age civilizations of the region. These dates suggest that the differentiation of the social systems in Afghanistan could have been driven by the emergence of the first urban civilizations. However, the dates suggested by BATWING should be treated with care, since BATWING does not model gene flow and differential assimilation of incoming migrations. These events could alter the time of split. However, it was previously shown that topologies and times of splits in the modal trees generated by BATWING are insensitive to in-migration [13], which leaves BATWING timing results insusceptible to in-migrations and invasions that might be expected to reduce the times of split [13]. On the other hand, the times of population splits for BATWING's modal trees are very susceptible to subsequent migration between those populations. This means that the 2 major waves of splitting could have occurred earlier, but since RM networks of the major haplogroups show limited gene flow between the ethnic groups and since the population structure suggested by MDS and Barrier correlate populations from the historically connected [2] Bronze Age sites to Pashtun and Tajik, BATWING suggested splits in Afghan populations at 4.7 kya (95% CI 2,775–7,725) are very probable. A previous study by Heyer et al conducted in Central Asia [40] have also estimated significantly older dates for the emergence of ethnic groups from what has been historically known. These older dates may be explained by the fact that This suggests that the ethnic groups could have resulted from a encompass fusion of different populations [40] or that ethnicities developed were established from anin already structured population(s).

BATWING's hypotheses model mutations and coalescent events, reflecting ancestral structures from which the current populations have emerged. Later expansions into the region would have assimilated the ancestral population, granting the Afghans distinctive genetics from the expanding source populations even though they shared general genetic features. This is evident in the Afghan Hazara and Afghan Uzbek who have always been associated with expanding Mongols and Turco-Mongols. Although we have found that at least third to half of their chromosomes are of East Asian origin, PCA places them between East Asia and Caucasus/Middle East/Europe clusters.

Historical expansions and invasions appear to have had differential contribution in shaping Afghanistan population structures. We have found limited genetic evidence of expansions previously thought to have left specific imprints in current populations.

The E1b1b1-M35 lineages in some Pakistani Pashtun were previously traced to a Greek origin brought by Alexander's invasions [48]. However, RM network of E1b1b1-M35 found that Afghanistan's lineages are correlated with Middle Easterners and Iranians but not with populations from the Balkans.

The Islamic invasion in the 7^th^ century CE left an immense cultural impact on the region, with reports of Arabs settling in Afghanistan and mixing with the local population [49]. However the genetic signal of this expansion is not clearly evident: some Middle Eastern lineages such as E1b1b1-M35 are present in Afghanistan, but the most prevalent lineage among Arabs (J1-M267) was only found in one Afghan subject. In addition, the three Afghans that identified their ethnicity as Arab, had lineages autochthonous to India.

We also note that three Hazara subjects belonged to haplogroup B-M60, which is very rare outside Africa. RM network shows that the subjects had a recent founding ancestor from East Africa, which could have been brought to Afghanistan through slave trade. This shows that the genetic ethnic boundaries have been selectively permeable, however the history of the rules of assimilation in this region over time are not yet clearly understood.

Language adoption and spread in Afghanistan also seem to have been a complex process. The Afghan genetic structure tends to correlate Hazara and Uzbek which belong to two different language families. Hazara, like Pashtun and Tajik, belong to the Indo-Iranian group of the Indo-European family, while the Uzbek language is in the Turkic family. The form of Turkic spoken by the Uzbek appears to be a direct descendent of an extinct Turkic language that was developed in the 15^th^ century CE [50]. It appears that the dominating genetics shared among Uzbek and Hazara split >1 ky prior to this date. Therefore, it is possible that language differences in Afghanistan reflect a more recent cultural shift.

In conclusion, Y-chromosome diversity in Afghanistan reveals major differences among its ethnic groups. However, we have found that all Afghans largely share a heritage of a common ancestral population that emerged during the Neolithic revolution and remained unstructured until 4.7 kya (95% CI 2,775–7,725). The first genetic structures between the different social systems started during the Bronze Age accompanied, or driven, by the formation of the first civilizations in the region. Later migrations and invasions to the region have been differentially assimilated by the ethnic groups, increasing inter-population genetic differences, and giving the Afghan a unique genetic diversity in Central Asia.

## Supporting Information

Figure S1 **Reduced median networks.** (A) C-M130, (B) R1a1a-M17, (C) E1b1b1-M35, and (D) B-M60 showing STR haplotype distributions among populations; area is proportional to haplotype frequency, and color indicates populations. Connecting lines represent putative phylogenetic relationships between haplotypes.
(TIF)

Table S1 Suggested origins of the main ethnic groups in Afghanistan.
(DOC)

Table S2 Y-chromosome haplogroups and haplotypes in 204 unrelated individuals from Afghanistan.
(XLS)

Table S3 Populations selected for this study.
(XLS)

Table S4 Y-chromosome haplogroups frequencies in Afghanistan's ethnic groups.
(XLS)

Table S5 AMOVA results. Comparing populations grouped according to their country or region of origin with populations grouped according to Barrier structures.
(DOC)

Table S6 BATWING topologies and dates with 95% confidence intervals of population splits derived from multiple combinations of population subsets.
(XLS)
