# Supplementary figures and images for: Afghanistan's Ethnic Groups Share a Y-Chromosomal Heritage Structured by Historical Events
Source: PLoS One. 2012 Mar 28;7(3):e34288. doi: 10.1371/journal.pone.0034288 (PMC3314501; doi:10.1371/journal.pone.0034288)

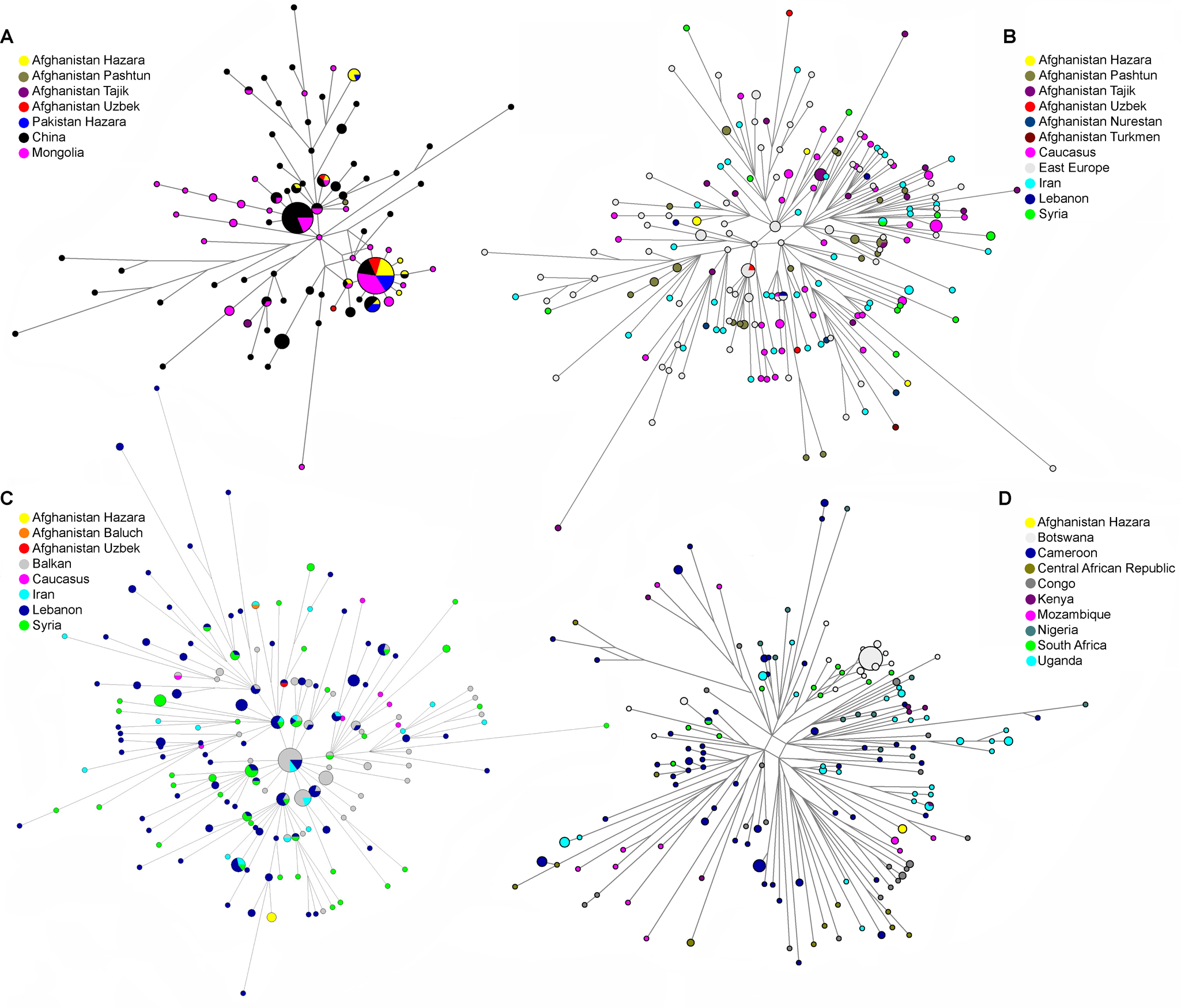

Supplement: Figure S1 — Reduced median networks. (A) C-M130, (B) R1a1a-M17, (C) E1b1b1-M35, and (D) B-M60 showing STR haplotype distributions among populations; area is proportional to haplotype frequency, and color indicates populations. Connecting lines represent putative phylogenetic relationships between haplotypes. (TIF) [file pone.0034288.s001.tif]
