# Supplementary material for: Afghanistan's Ethnic Groups Share a Y-Chromosomal Heritage Structured by Historical Events
Source: PLoS One. 2012 Mar 28;7(3):e34288. doi: 10.1371/journal.pone.0034288 (PMC3314501; doi:10.1371/journal.pone.0034288)
Supplement: Table S1 — Suggested origins of the main ethnic groups in Afghanistan. (DOC) [file pone.0034288.s002.doc]

|  |  | **Suggested Origin** | |
| --- | --- | --- | --- |
| **Ethnic Group** | **Location in Afghanistan** | **Oral history** | **Genetic finding of this study** |
| **Pashtun** | South,  south west,  and west | At least three main suggested origins:  Jewish: Israelite tribes that were exiled by the Assyrian Empire 2,700 years ago.  Greek: remnants of Alexander’s army.  Rajput: mainly found in north India. | Shows genetic affinity to north and west India. Pashtun split from the rest of the Afghans 4.7 kya during the rise of the area’s first civilizations at the Indus Valley and Bactria-Margiana. The main lineage in Pashtun has the oldest coalescent time 14 kya in the Indus Valley. |
| **Tajik** | North east, center, and west | Believed to be of Iranian origin. The word “Tajik”, means non-Turk in Turkic. | Like Pashtun, Tajik shows genetic affinity to north and west India. Tajik haplotypes are also very similar to Iranians especially from Ardabil. |
| **Hazara** | Center | Believed to be remnants of Genghis Khan’s soldiers. Mongol troops were left behind in detachments of a thousand and Hazar in Persian means  “thousand”. | 30% of the lineages in Hazara were previously associated to Genghis Khan and his Mongol army. However, Hazara have also genetic elements of populations that have probably inhabited the area before the Mongol invasion, making them very distinct from the Mongols themselves. |
| **Uzbek** | North | Turkic nomadic herders that settled during the Turko-Mongol invasion to Central Asia. | 40% of the lineages in Uzbek were previously associated to Genghis Khan and his Mongol army. But also like Hazara, Uzbek are very distinct from Mongols probably due to mixing with endogenous populations. |

**Table S1: Suggested origins of the main ethnic groups in Afghanistan.**
