# Supplementary material for: Afghanistan's Ethnic Groups Share a Y-Chromosomal Heritage Structured by Historical Events
Source: PLoS One. 2012 Mar 28;7(3):e34288. doi: 10.1371/journal.pone.0034288 (PMC3314501; doi:10.1371/journal.pone.0034288)
Supplement: Table S5 — AMOVA results. Comparing populations grouped according to their country or region of origin with populations grouped according to Barrier structures. (DOC) [file pone.0034288.s006.doc]

**Table S5: AMOVA results.** Comparing populations grouped according to their country or region of origin with populations grouped according to Barrier structures.

| **Groups** |  | $V_{a};F_{CT}$ **Among Groups** | $V_{b};F_{SC}$ **Within Groups** | $V_{c};F_{ST}$ **Within Populations** |
| --- | --- | --- | --- | --- |
| **Populations grouped by country or region** | Value | 1.097;0.071 | 0.759;0.053 | 13.476;0.121 |
|  | % variation | 7.16 | 4.95 | 87.89 |
|  | p-value | <0.001 | <0.001 | <0.001 |
| **Populations grouped according to Barrier** | Value | 1.598;0.104 | 0.350;0.025 | 13.301;0.127 |
|  | % variation | 10.48 | 2.30 | 87.22 |
|  | p-value | <0.001 | <0.001 | <0.001 |
